# Parental experience and information needs following a positive newborn screening test-based neonatal diagnosis of a chronic condition: an integrative review

**DOI:** 10.1016/j.ijnsa.2026.100646

**Published:** 2026-07-30

**Authors:** Patricia Luck, Jana Pachlopnik -Schmid, Rahel Naef, André Fringer, Susan Kirk, Anna-Barbara Schlüer

**Affiliations:** a Division of Immunology University Children’s Hospital, Zurich, Switzerland; b Children’s Research Centre University Children’s Hospital, Zurich, Switzerland; c University of Zurich, Zurich, Switzerland; d Institute for Implementation Science in Health Care, Faculty of Medicine, University of Zurich, Zurich, Switzerland; e Centre of Clinical Nursing Science, University Hospital Zurich, Zurich, Switzerland; f Institute of Nursing, School of Health Sciences, ZHAW Zurich University of Applied Sciences, Winterthur, Switzerland; g School of Health Sciences, The University of Manchester and Manchester Academic Health Science Centre (MAHSC), Manchester, UK

**Keywords:** Neonatal screening, Parents’ experiences, Parents’ information needs, Integrative Review

## Abstract

**Background:**

Preventive routine Newborn Screening for rare, chronic conditions, such as metabolic diseases, can lead to unexpected and sudden diagnoses within the first weeks of neonatal life. A child’s chronic illness diagnosis is distressing for parents, who must learn to manage the condition at home and have high information needs during this time. Evidence on parental experiences of receiving a diagnosis following a positive Newborn Screening test and on their unmet needs is limited.

**Objective:**

To synthesize research knowledge on experiences and information needs of parents following positive Newborn Screening -based neonatal diagnosis of a chronic condition.

**Information sources:**

Electronic databases of information sources (PubMed, Livivo, Cochrane, CINAHL, and Embase) were searched.

**Methods:**

An integrative literature review was conducted using Whittemore and Knafl's methods. Search, selection and inclusion process was reported following PRISMA guidelines. Studies were included if they focused on experiences and information needs of parents after the diagnosis of a chronic condition following a positive Newborn Screening result. Publications published from inception until June 10th 2025 were included. Included articles were individually appraised for quality according to the Equator Guidelines: COREQ-32, STROBE, and JARS Mixed Tables. A Constant Comparative Analysis was used for data analysis and synthesis.

**Results:**

The systematic search yielded 3416 hits, of which 14 articles were included; these included eleven qualitative, one quantitative, and two mixed-method design studies. Analysis of the included articles revealed a multi-level process that parents go through. Findings were organized into two main categories: ‘Feeling Overwhelmed’ and ‘Becoming Active’. Two subcategories described parental experiences: ‘feeling shocked and going through many challenging emotions’, ‘urge to search for information to appraise severity’, and ‘focusing on actionable elements’. Moreover, three subcategories referred to parental information needs: ‘Needing to understand with a limited ability to absorb’ followed by ‘Specialized Health Care Professional (HCP) as point of contact and the preferred information source’, which consists of: ‘Learning about practical aspects of condition management at home’, and ‘Learning about how to manage as a family’.

**Conclusions:**

This review provides insights into parents’ experiences and information needs following their neonate’s diagnosis of a chronic condition after a positive Newborn Screening result. Parents require condition-specific information, psychosocial support from specialized HCPs, and accessible materials in various formats. Guidelines based on these findings can improve information provision and support for all families regardless of socioeconomic status or educational level, thereby enhancing access to quality essential healthcare services and health equity.

**Registration:**

Not registered.

**What is already known**

• The unexpected diagnosis of Newborn Screening impacts parents and the whole family
• Time between a positive Newborn Screening and confirmation is worrying for parents

Alt-text: Unlabelled box dummy alt text

**What this paper adds**

• Personalized information helps parents to improve their family condition management
• Diagnoses of rare diseases by Newborn Screening lead to a need to appraise severity
• While parents search the internet, HCPs are the preferred and trusted source

Alt-text: Unlabelled box dummy alt text

## 1 **Background**

Newborn Screening enables the detection of treatable but not clinically evident diseases during the neonatal period. As the number of screening tests has increased, so too has the number of conditions included in Newborn Screening programmes (Fabie et al., 2019). Newborn Screening is typically incorporated within a public health programme and is nationally regulated (Currier and Puck, 2021). Consequently, disorders included vary between countries and are predominantly established in high-income countries (Loeber et al., 2021).

An Newborn Screening panel consists of on-site tests and blood analysis conducted within the first few days after birth (Fabie et al., 2019). If a neonate tests positive for a condition, parents are notified about the positive test, potential diagnosis, and treatment (Fabie et al., 2019). The method of notifying parents of a positive Newborn Screening test result is subject to variation by country and even region, some possible options are: phone call, medical appointment or online parental portal (El-Hattab et al., 2018). Shortly thereafter, an appointment is set for diagnostic testing to ensure treatment is initiated in timely manner (Fabie et al., 2019). This pre-appointment period is reported to be distressing for most parents (White et al., 2021). If the diagnostic test does not confirm the condition, the Newborn Screening result is considered a false positive, and the neonate does not require further treatment (El-Hattab et al., 2018). Similarly, no treatment is required if carrier status is identified through Newborn Screening, which is the case in some countries (Farrell et al., 2020). Although parents who have received a false positive result or a carrier status for their child do require specialized information, they are not required to learn how to manage the condition (Farrell et al., 2020; White et al., 2021). However, if a condition is confirmed, a diagnosis of a chronic rare disease is made (Fabie et al., 2019).

The diagnostic course is unexpected and distinct following a positive Newborn Screening test than one based on symptoms (Brockow and Nennstiel, 2019; Domaradzki and Walkowiak, 2024). Parents often perceive their neonate to be healthy (Chudleigh et al., 2020), as there are often no visible symptoms (White et al., 2021). Consequently, the diagnosis is challenging for parents to understand (White et al., 2021). The diagnosis and the period immediately following this have been found to be the most distressing time for parents (Thomas et al., 2023). They experience a sense of loss regarding their future (Pelentsov et al., 2015) and identity (Smith et al., 2015). Parents must manage their neonates’ condition within the home setting (Fabie et al., 2019). Parents have a heightened need for information regarding the condition, its treatment, and impact on their child’s and the family’s future (Pelentsov et al., 2015; Smith et al., 2015; White et al., 2021).

The information needs of parents appear to evolve over time (Hummelinck and Pollock, 2006). Insufficient information negatively affects parents (Pelentsov et al., 2015). Studies suggest that parents who receive support and encouragement from health care professionals (HCPs) find it helpful in acquiring the skills to manage their child’s chronic condition and adapt to their new role as caregivers (Loura et al., 2024; Nygård and Clancy, 2018), potentially leading to a reduced caregiver burden (Shoghi et al., 2019). A study about the adjustment process following the diagnosis of congenital anomalies revealed that the provision of information about the condition had a positive influence on the adaptation process, with general improvements in parental adjustment after six months (Fonseca et al., 2014).

In summary, parents of an affected neonate carry a high emotional burden and have different needs. However, there is currently a lack of research on parents’ experiences with this specific diagnostic course and their resulting needs. Current research on this topic includes studies of parents of children with one condition (i.e.: cystic fibrosis (Brockow and Nennstiel, 2019) or from one medical field (i.e., metabolic conditions, (Buchbinder and Timmermans, 2012). In addition, literature reviews have been published that focus on the medical aspects of Newborn Screening or attitudes towards it (El-Hattab et al., 2018; Tluczek et al., 2022). To the best of our knowledge, there is only one review of studies that included parents of newborns with different conditions diagnosed after a positive Newborn Screening test (White et al., 2021). White et al. (2021) provide an in-depth analysis of parents’ experiences until a confirmed diagnosis, but not beyond that. Further information is required on family management of neonates with the diagnosed conditions at home, their experiences of the vulnerable period of the first six months after diagnosis, and their information needs. The term family management refers to the way which families cope with a child’s chronic condition after it has been diagnosed, and incorporation of condition management into their daily lives over time (Knafl et al., 2012). In particular, parental information needs should be addressed by HCPs to support families in managing their child’s condition. A more comprehensive understanding, acquired through specific research, will assist HCPs in developing personalised information and support programmes for these families. Therefore, the aim of this review is to synthesize research knowledge on the experiences and information needs of parents after a neonatal diagnosis of a chronic condition following positive Newborn Screening.

## 2 Methods and design

We undertook an integrative review (IR) following the methodology of Whittemore and Knafel (2005), because it enables the inclusion of all relevant literature to answer the research question, regardless of the methodology. The IR allows the synthesis of data from experimental, non-experimental, qualitative, and theoretical articles (Whittemore and Knafl, 2005). So a broad range of evidence sources can be included, leading to a comprehensive understanding of the complex phenomena being investigated. This approach best suits the research question by allowing inclusion of diverse evidence through an exploratory, inductive method that recognizes patterns combining qualitative and quantitative aspects. The main steps of an IR are problem identification, literature search, data evaluation, data analysis, and presentation (Whittemore and Knafl, 2005). No review protocol was registered.

As no specific reporting guidelines for IRs exist, and IR follows a systematic approach to search literature we report the search based on PRISMA guidelines combined with the methodology paper by Whittemore and Knafl (2005).

### 2.1 IR step 1: problem identification

A positive Newborn Screening result that leads to the diagnosis of a chronic condition is a unique and sudden diagnostic course. To gain a more comprehensive understanding of parents’ experiences and information needs throughout this diagnostic pathway, studies examining the conditions regularly tested in Newborn Screening programs were included and compared. Parents are very vulnerable in the period after a positive Newborn Screening result leads to a diagnosis. Therefore, the focus was on the first 6 months. A systematic, integrative literature review was conducted to answer the following **research question**:

What are the experiences and information needs of parents during the first 6 months after the diagnosis of a chronic condition following a positive blood-spot Newborn Screening test of their neonate, with regard to family management of the condition?

The **eligibility criteria** were defined based on the PEO framework (Population, Exposure, Outcome) (Moola et al., 2015). See Table 1.

**Table 1:** Eligibility criteria based on PEO framework.

Criteria	Inclusion	Exclusion
Population	- Parents of a neonate	-
Exposure	- Neonate diagnosed with a chronic condition after a positive blood-spot Newborn Screening result	- Effects of a false positive Newborn Screening result - Unclear diagnosis - Degenerative conditions without treatment options - Genomic sequencing
Outcome	- Experience and impact during the first few months after diagnosis (until approximately 6 months after) - Resulting information needs and educational needs - coping - Family management - Family-centred care - support	- Attitudes towards Newborn Screening - Inclusion or exclusion of new conditions not established in a country yet - Consent for Newborn Screening - Impact of carrier status - People with traits - Medically underserved areas / countries - Experience of Newborn Screening process only until first appointment - Experience of treatment - Medical outcomes - Treatment outcomes - Secondary research, editorials, protocols, opinion papers, conference, reviews (to avoid including results from articles twice), books, dissertations - Anonymous sources like online posts - Cost effectiveness
Filters	- Studies published in English	- Non-English papers

### 2.2 IR step 2: literature search

The topic of parents’ experiences and information needs may be related to several healthcare areas. The following five search engines in the field of health care science were used as **information sources**: PubMed, CINAHL, Cochrane, Embase, and Livio. These five databases were chosen for comprehensive coverage: PubMed, Embase, and Cochrane for healthcare topics; CINAHL for nursing and allied health focus; and Livivo for interdisciplinary health topics. No preliminary search was performed.

The search was originally performed December 22nd 2023, and updated on June 10th 2025 by combining “experience” and “information needs” with Boolsche Operator OR rather than AND based on a peer reviewer’s suggestion (PL).

The search terms for the **search strategy** were selected based on the PEO framework. The detailed search strategy is presented in Appendix A. The first and last authors discussed the search strategy, consulted an information specialist, and accordingly agreed upon the search strategy. Each database was searched individually, and the search strategy was adapted if necessary.

For the **selection process,** studies were screened by title, abstract, and full text successively against the eligibility criteria by the first author, and uncertainties in the selection process were discussed with the last author. No software was used for the selection process. Results of the search, duplicate records, and selection after title screening were extracted and saved; after abstract and full-length article screening, a list was created with the excluded studies and the corresponding reason for exclusion.

### 2.3 IR step 3: data evaluation, critical appraisal of individual sources of evidence

The included studies were critically **appraised for their quality** based on the EQUATOR guidelines according to their design. A quality score was created for each study, with one point given, for each fulfilled item of the guideline, ranging from 0 to 32 for COREQ-32, 0 to 22 for STROBE and 0 to 33 for JARS. For a better understanding of the quality appraisal and comparison of the strength of evidence, the total number of items in each guideline was divided into four categories: poor, fair, good, and excellent. It was predetermined that articles with poor quality would be excluded. The following guidelines were applied:

− For qualitative studies, the COREQ-32* and categories with a range of numbers of items were poor (1- 8), fair (9 – 16), good (17 – 24), excellent (25 - 32)
− for quantitative, STROBE**, and categories with a range of items: poor (1 – 5), fair (6 – 11), good (12 – 16), and excellent (17–22).
− for mixed methods, JARS*** guidelines, and categories with a range of items: poor (1 – 8), fair (9 – 16), good (17 – 25), and excellent (26–33).

We used reporting guidelines to assess quality, awarding points only when items demonstrated good quality implementation rather than merely counting their mention. Two researchers (PL, BW, and ABS) independently assessed each study, then discussed individual appraisals to reach a joint final decision.

### 2.4 IR step 4 and 5: data analysis and presentation

The full-text results of all included studies were imported into MAXQDA (version 24.3.0) for **data collection and data extraction** (PL). The **data items** were based on eligibility criteria (Table 1). The first author (PL) summarised the included studies. A Constant Comparative Analysis (CCA) (Glaser, 1965) was conducted for the **synthesis of results**, as suggested by Whittemore and Knafl (2005). This method is suitable for the systematic integration of heterogeneous data while following a rigorous process. CCA follows an iterative process in which data and codes are constantly compared and further synthesized into subcategories and categories. This approach was applied to the results of the included studies to answer the underlying research question (PL). Code-Matrix-Browser, a visual aid tool for MAXQDA, was created to support this procedure. A model was developed to present the conclusions and synthesis (Fig. 2).

## 3 Results

### 3.1 Characteristics of the studies

**Fig. 1:**
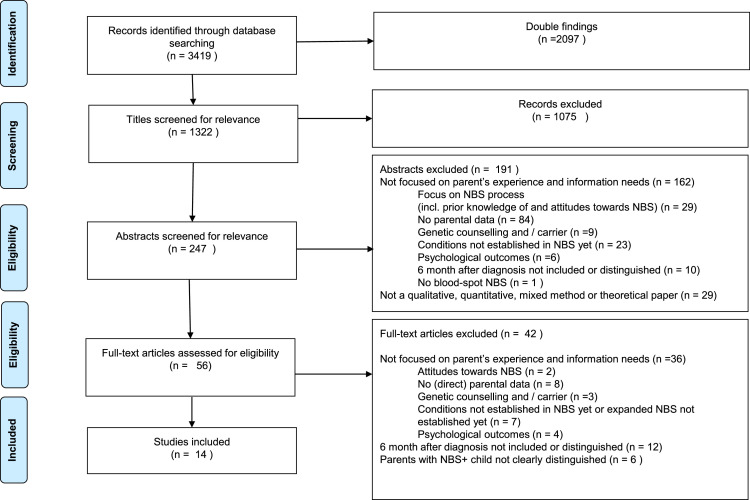
Literature review flow-chart.

The systematic literature search identified 3416 studies (Fig. 1). Consequently, 14 articles were included in this review. All 14 studies were rated as good to excellent quality (for individual quality appraisal and categorised levels, see Table 2). Each of the 14 studies included information relevant to the research question. Information not relevant to the aims of this study was excluded from the synthesis. Articles on topics of importance for this review are summarised in Table 2.

**Table 2:** Summary of included studies.

First Authors, Year Country	Design	Aim	Participants and child’s condition	N	N of mothers and fathers	Data collection	Data analysis	Summary of results	Quality Appraisal
Qualitative Studies:									
Boyse et al. 2014 USA	A qualitative descriptive design (Sandelowski, 2000)	Characterize early parent caregiver experiences and needs in CAH with focus on contextual factors that can be modulated through education and support delivered by healthcare providers.	Parents of neonates and young children identified by Newborn Screening with congenital adrenal hyperplasia (CAH)	6	4 mothers, 2 fathers (total of 4 families)	Semi-structured telephone interviews	Conventional Content Analysis	Three main themes: • Communicating health information • Unmet needs, • Social support	Good * = 20
Carpenter et al. 2018 UK	Qualitative Design	Explore the lived experience of parenting a child with PKU in the first two years.	Parents with children under two years old with phenylketonuria (PKU)	7	6 mothers 1 father	Semi-structured interviews	interpretative phenomenological analysis (IPA: Smith and Osborn, 2008)	Three subordinate themes: • Control • Striving for normality • Acceptance of PKU as a continuum	Good * = 22
Chudleigh et al. 2016 UK	Qualitative Design	Gain insight into parents' experiences, explore the effects of the result on the family, the impact on the parents' relationships with health professionals, and consider alternative ways of sharing positive Newborn Screening results with parents.	Parents of infants 12 months or younger diagnosed with Cystic Fibrosis (CF) or Sickle Cell Disease (SCD) via the Newborn Screening program were invited to participate. *SCD results excluded from this review*	12	12 mothers 10 fathers	Semi-structured interviews	Core principles of grounded theory	Themes 1: prior knowledge of the condition Theme 2: Receiving the initial positive Newborn Screening result Theme 3:Reactions to the positive Newborn Screening result Theme 4: sharing the result with others Theme 5: impact of the screening process on parental relationships Theme 6: Future support strategies	Good *= 20
DeLuca et al. 2011 USA	Qualitative Descriptive Design	Create a description of parents' experiences of their infants' evaluations for rare metabolic disorders as they unfold in real time.	English-speaking parents with abnormal metabolic results	44	30 mothers 14 fathers (total of 30 families)	Semi-structured, open-ended interviews	Qualitative content analysis	Categories: • Parents response to initial call • Internet use • Metabolic treatment centre visit • Waiting for test results • Equivocal results • Parents’ views on newborn screening	Excellent *= 25
Howley et al. 2024 UK	Qualitative Design	The following narrative highlights information needs as presented by families of infants with confirmed or suspected athymia, specifically during the diagnostic period following an abnormal Newborn Screening result.	Parents of children with athymia diagnosed following a positive Newborn Screening, 9 participated, 5 declined	9	9 families	Patient involvement and engagement project with focus group interviews	Not specified	Five categories: • Initial Newborn Screening results • Differential diagnoses • Diagnostic pathway in genetically undefined T-lymphocytopenia • Availability of information on athymia and thymus transplant Access to support networks.	Good *=17
Jessup et al. 2016 Australia	Qualitative Design	Understand parents' experiences of initial education following their infant's diagnosis with CF following Newborn Screening.	Parents of a child newly diagnosed with CF were invited to participate in the 3-day education	10	7 mothers 3 fathers (total of 7 families)	Interviews	Van Manen’s phenomenological approach With selective reading	Stages: • Initial response • People, process and pragmatics • Reassurance and Empowerment	Excellent *= 26
Kutsa et al. 2022 USA	Qualitative Design	Understand parents many uncertainties emanating from their SCID journey and identify ways parents find to cope with them.	Parents of a child with Severe Combined Immunodeficiency (SCID) or SCID-like condition identified through Newborn Screening	26	21 mothers 5 fathers	Semi-structured virtual interviews	Inductive content analysis	Coping strategies (behavioural, cognitive, affective) following SCID trajectory: Coping in the diagnosis stage, Coping in the pre-treatment stage Coping in the treatment stage Coping in the post-treatment stage Coping in the new normal stage	Good *= 23
Nielsen et al. 2022 Denmark	Qualitative Design	Explore everyday life experiences of parents whose children had been diagnosed with CF at the time of diagnosis and in the initial months after the diagnosis was made.	Parents of a child diagnosed with Cystic Fibrosis (CF) in Newborn Screening	29	15 mothers 14 fathers (total of 15 families)	Narrative interviews combined with field observation of consultation	Analysis after Brinkmann and Kvale	Three main themes: • Receiving the diagnosis • The first meeting in the CF centre • The new everyday life and the future	Good *= 21
Priddis et al. 2010 Australia	Qualitative Design	Examines paternal responses to a diagnosis of CF in their infant.	Fathers of children with CF: Diagnosed at least 6 months prior to research participation, only having one child with CF, part of an intact family, parents understand English.	15	15 fathers	semi-structured interviews at the participants home (13) or by phone (2)	Analysed after Smith, 2001	Four key themes: • Emotional adjustment to diagnosis • Life after the CF diagnosis • Changes in the family systems Relationship with hospital	Good *= 19
Pruniski et al. 2018 USA	Qualitative Design	Examines the effect of PD diagnosis via Newborn Screening on parents and families, in (particular the extend to which concerns regarding Newborn Screening for late-onset PD are being realized).	Parents of a child with Pompe’s Disease (PD) (Late-onset PD parents excluded for this review)	9	(9 mothers) 3 mothers of children with Early onset 0 fathers	In-depth interviews	Deductive and inductive coding, grounded theory	Themes: • Receiving positive Newborn Screening • Initial communication of results, • Uncertainty • Emotional reactions, • Coping strategies, • Family dynamics/ Medicalization, • Insurance & financial concerns, • Reproductive benefit & cascade testing, • Overall opinion of Newborn Screening for PD, Advice for other families & HCPs	Good *= 17
Raspa et al. 2024 USA	Qualitative Design	Exploring the types of uncertainties experienced by parents of a child with SCID diagnosed through Newborn Screening.	Parents of a child with Severe Combined Immunodeficiency (SCID) or SCID-like condition identified through Newborn Screening	26	21 mothers 5 fathers	Semi-structured in-depth, virtual interviews	Inductive content analysis	Organized around the issues of uncertainty experienced by parents: Scientific, Practical, Personal, Existential uncertainties Reaction to uncertainty	Good *= 19
Quantitative Studies:									
Edwards et al. 2018 Australia	Quantitative Cross-sectional Design	Investigate the information needs, priorities, and information-seeking behaviours of parents of infants newly diagnosed with CF following Newborn Screening by piloting the “Care of Cystic Fibrosis Families Survey”.	Parents of Cystic Fibrosis (CF) patients	66	No participant’s characteristics	The Care of Cystic Fibrosis Families Survey,	Descriptive Statistics and open-ended questions grouped and summarized	N = 26 response rate 39.4% Parents from 2 CF centres received an educational period ranging from 3 to over 5 days. Rating of components of educational period as “essential to know”: Antibiotics, hospital visits, medication, Caring for their child (daily routines at home & mealtimes) (n = 25, 96.2%) Organization of initial education session: Majority received right amount of information (n = 19, 73.1%), Preferred time period of delivery: few days/ one week (n = 11, 42.3%), Open end questions to preferred source of information: Face to face meetings (n = 14),	Excellent ** = 17
Mixed Method Studies:									
Lebensburger et al. 2015 USA	Mixed Method Design	Goals were to identify what topics are pertinent to parents at their initial sickle cell clinic visit, how and when they would like to learn about the disease, and what motivates them to seek health information. In addition, the project sought to evaluate the perceived usefulness of the education materials used at the UAB clinic.	Sequential exploratory mixed method research: In-depth qualitative semi-structured interviews, a survey was developed on qualitative results and conducted Qual: 8 parents of an infant with sickle cell anaemia diagnosed following a pos. Newborn Screening, Quan: 22 parents	Qual: 8 Quan: 22	Qual: 7 mothers 1 father Quan: Not specified	Sequential exploratory mixed method Qual: In-depth semi-structured interviews, Quan: Development of a survey based on qualitative results and conducted	Qual: Constant Comparative Analysis Quan: Not specified	Two major themes: • Parental fear of sickle cell anaemia Trust in health sources of health information	Good ***=33
Raspa et al. 2020 USA	Mixed Method Design	Assess experiences of parents of a child with SCID, from diagnosis through post-treatment, informational and emotional support needs and what the desired points in time to receive information and support. Assess the differences in informational and emotional support needs by subgroups and parents rating of the available SCID-related materials and their preferred sources and formats.	7 parents of a child diagnosed with SCID through Newborn Screening participated in a journey mapping event (qual) And 76 completed the survey.	76 7	Survey: 66 mothers 10 fathers Interviews: 7 parents	In-depth interviews And online survey	Journey mapping	Five stages parents experience: • Diagnosis, • Pre-Treatment • Treatment • Post-Treatment The New Normal	Good ***= 20

Of the 14 studies, 11 had a qualitative design (Boyse et al., 2014; Carpenter et al., 2018; Chudleigh et al., 2016; DeLuca et al., 2011; Howley et al., 2024; Jessup et al., 2016; Kutsa et al., 2022; Nielsen et al., 2022; Priddis et al., 2010; Pruniski et al., 2018; Raspa et al., 2024), 1 had a quantitative design (Edwards et al., 2018), and 2 had a mixed-methods design (Lebensburger et al., 2015; Raspa et al., 2020); there were no studies with theoretical data. Studies were conducted in the United States of America (Boyse et al., 2014; DeLuca et al., 2011; Kutsa et al., 2022; Lebensburger et al., 2015; Pruniski et al., 2018; Raspa et al., 2020, 2024), United Kingdom (Carpenter et al., 2018; Chudleigh et al., 2016; Howley et al., 2024), Australia (Edwards et al., 2018; Jessup et al., 2016; Priddis et al., 2010), and Denmark (Nielsen et al., 2022). The studies focused on 1 condition, 2 conditions or a category of conditions (i.e., metabolic diseases). The conditions were as follows: metabolic diseases in general (DeLuca et al., 2011), and single metabolic conditions such as Pompe disease (Pruniski et al., 2018), congenital adrenal hyperplasia (Boyse et al., 2014), phenylketonuria (Carpenter et al., 2018), sickle cell disease (SCD) (Lebensburger et al., 2015), and cystic fibrosis (CF) (Edwards et al., 2018; Jessup et al., 2016; Nielsen et al., 2022; Priddis et al., 2010). One study included parents of children with CF and SCD (Chudleigh et al., 2016). An additional group were severe combined immunodeficiencies (SCID) (Kutsa et al., 2022; Raspa et al., 2020, 2024) and athymia (Howley et al., 2024).

The three studies on SCID were undertaken by the same research group; two studies (Kutsa et al., 2022; Raspa et al., 2024) appeared to be from the same dataset. The third study (Raspa et al., 2020) was undertaken earlier, and a different dataset was used. Two studies on CF were conducted by the same research group but with a different dataset (Edwards et al., 2018; Jessup et al., 2016).

### 3.2 Overarching process

**Fig. 2:**
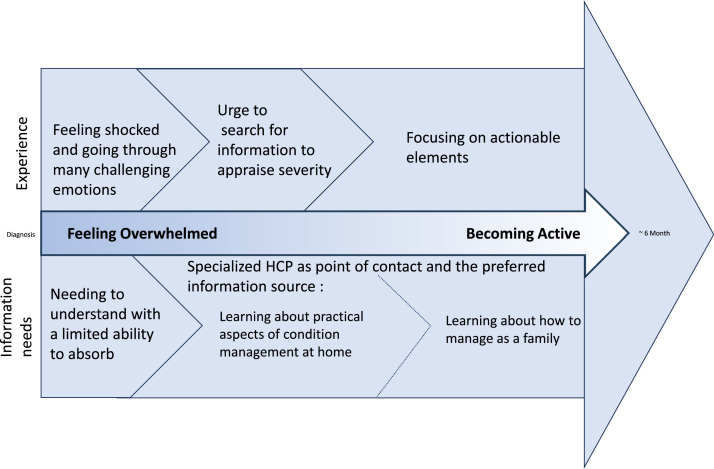
Parents’ progression after a positive NBS result leading to the diagnosis of a chronic illness.

We identified a core process that progressed over time from feeling overwhelmed to becoming active (Fig. 2), spanning the first 6 months towards the future. The process consists of two main categories explaining a development that parents seem to undergo in learning to manage the condition as a family: starting from ‘Feeling overwhelmed’ (category 1) towards ‘Becoming active’ (category 2). “Feeling overwhelmed” appears to be a more passive state, due to parents being in shock. However, nearly simultaneously, parents start to take small steps towards activity, which increases over time and is mapped out in “Becoming active”. All steps of activity are necessary to enable family management of the condition at home and appear to serve as a strategy to handle the situation. The two main categories are each divided into subcategories pertaining to either experience or information needs.

### 3.3 Category 1: feeling overwhelmed

After the parents received information about their children’s positive Newborn Screening results, they felt overwhelmed by the situation. This finding was reported in five studies (Boyse et al., 2014; Jessup et al., 2016; Kutsa et al., 2022; Raspa et al., 2020, 2024). Additionally, parents described feeling overwhelmed by the explanations they received from the HCP (DeLuca et al., 2011) or by what they found on the Internet (Edwards et al., 2018). In one article, parents mentioned feeling overwhelmed by the sudden demands connected to managing their child’s condition at home (Carpenter et al., 2018). The category ‘Feeling overwhelmed’ indicates the starting point of the process and can be described as a more passive shock state. This category comprised two subcategories. One parental experience subcategory is ‘feeling shocked and going through many challenging emotions’. The parents’ information needs subcategory is dominated by a ‘needing to understand with a limited ability to absorb’.

#### 3.3.1 Subcategory: feeling shocked and going through many challenging emotions

Ten studies described parents being shocked and experiencing many strong and conflicting emotions simultaneously (Carpenter et al., 2018; Chudleigh et al., 2016; DeLuca et al., 2011; Jessup et al., 2016; Kutsa et al., 2022; Lebensburger et al., 2015; Nielsen et al., 2022; Priddis et al., 2010; Pruniski et al., 2018; Raspa et al., 2020). These emotions include fear (Jessup et al., 2016; Kutsa et al., 2022; Lebensburger et al., 2015; Nielsen et al., 2022; Priddis et al., 2010; Pruniski et al., 2018; Raspa et al., 2024), sadness,(Carpenter et al., 2018; Chudleigh et al., 2016; Lebensburger et al., 2015), and confusion (Kutsa et al., 2022). Parents explained this ‘*diagnosis-induced shock’* (Jessup et al., 2016) with a frequent statement as ‘*their world was turned upside down*’ (Chudleigh et al., 2016; Nielsen et al., 2022; Raspa et al., 2020). Other parents have compared their experiences with the occurrence of death (DeLuca et al., 2011). In a citation from Chudleigh et al. (2016), the father recounted:

*‘It hit me like a ton of bricks. I picked [Baby] up and just went next door with her and just, like, cried my eyes out really well if truth be known and, ‘Why us?’’*

#### 3.3.2 Subcategory: needing to understand with a limited ability to absorb

Thirteen studies reported that parents need to understand the situation as they are suddenly confronted with what is to them an unknown chronic condition, too (Boyse et al., 2014; Carpenter et al., 2018; Chudleigh et al., 2016; DeLuca et al., 2011; Howley et al., 2024; Jessup et al., 2016; Kutsa et al., 2022; Lebensburger et al., 2015; Nielsen et al., 2022; Priddis et al., 2010; Pruniski et al., 2018; Raspa et al., 2020, 2024). The time before they began to grasp the extent of the impact that the condition had on their family life was described as the most challenging and scariest period in the process (Boyse et al., 2014). Concurrently, parents had a number of questions regarding the condition and its cause, which they perceived as cause of uncertainty (Kutsa et al., 2022). Parents described the need to understand what the chronic condition meant for the health of their child (Carpenter et al., 2018; Jessup et al., 2016; Kutsa et al., 2022), and tried to understand the consequences for their child, themselves, their family, and their future (Boyse et al., 2014; DeLuca et al., 2011; Jessup et al., 2016; Lebensburger et al., 2015; Priddis et al., 2010; Raspa et al., 2024). Jessup et al. (2016) phrased this, trying to make sense of the situation as *‘embarking on their new role of parents of a child with a chronic illness’*.

This has to be understood in contrast to their limited ability to absorb information provided to them. The emotional shock that parents experience due to an unexpected diagnosis was also said to have led to a state of impaired receptivity (Boyse et al., 2014; DeLuca et al., 2011; Jessup et al., 2016). During their first notification or medical appointment with specialists, parents expressed difficulties in absorbing and understanding what they had been told (DeLuca et al., 2011; Jessup et al., 2016). It was pointed out that, owing to the emotional impact of the situation on them, their capability to process and remember information later was reduced (Boyse et al., 2014). One parent explained this in DeLuca et al. (2011).

*‘I couldn’t even comprehend anything that she said. I couldn’t even function at the moment she was telling me.’*

Other parents mentioned that they were trying to focus on information that gave them hope (Jessup et al., 2016).

### 3.4 Category 2: becoming active

Following a period in which helplessness and disorientation prevailed, across studies, parents reported how they became more active in managing life with a chronically ill neonate. Six studies reported that parents were driven by the need to understand the impact of their child’s condition as a first step towards action (Carpenter et al., 2018; Jessup et al., 2016; Kutsa et al., 2022; Lebensburger et al., 2015; Priddis et al., 2010; Pruniski et al., 2018). Parents finding an active mode of living with their child’s condition, it appeared to be an important managing mechanism that helped them go through the first period of strong emotions (Kutsa et al., 2022) and gave them a sense of control (Carpenter et al., 2018). In addition, parents described being able to break through circling thoughts by shifting their focus to taking action (Pruniski et al., 2018).

These parental experiences were described in the two subcategories: ‘urge to search for information to appraise severity’ and ‘focusing on actionable elements’. Parents’ information needs were also specified by the subcategory ‘Specialized HCP as point of contact and the preferred information source’, which is comprised of two sub subcategories—‘Learning about practical aspects of condition management at home’—followed by ‘Learning about how to manage as a family’.

#### 3.4.1 Subcategory: urge to search for information to appraise severity

Twelve studies reported that parents turned to the Internet for information (Boyse et al., 2014; Chudleigh et al., 2016; DeLuca et al., 2011; Edwards et al., 2018; Howley et al., 2024; Jessup et al., 2016; Kutsa et al., 2022; Lebensburger et al., 2015; Nielsen et al., 2022; Pruniski et al., 2018; Raspa et al., 2020, 2024). In one of these studies, a minority of the participating parents decided to conduct research online (DeLuca et al., 2011). Four articles stated that all participants accessed the Internet (Boyse et al., 2014; Chudleigh et al., 2016; Edwards et al., 2018; Nielsen et al., 2022). One article described searching the internet as a common next step after receiving the initial notification, such as a phone call (Raspa et al., 2024). Four studies declared that parents were advised by the HCP not to search online, but they still did so (Chudleigh et al., 2016; DeLuca et al., 2011; Kutsa et al., 2022; Raspa et al., 2024).

The time point mentioned for the online search was mostly between the notification giving the parents the Newborn Screening results and before their first appointment at the clinic.

Another time point for the online search was when parents waited for a diagnosis after a positive Newborn Screening result (Kutsa et al., 2022).

An online search was characterised as a personal quest for information to understand and trying to assess where their child was on a *“severity spectrum”*(Howley et al., 2024; Jessup et al., 2016; Lebensburger et al., 2015; Priddis et al., 2010). As a parent explained in DeLuca et al.(2011):

*‘We were furiously looking at the internet, trying to figure out what this was!’*

In one article, conducting an online search was described as a consciously active approach and a strategy for parents to brace themselves for the first consultation and prepare questions (Jessup et al., 2016). Alternatively, the online search was seen as adding information to what they heard and helping them ask additional questions (Edwards et al., 2018). Many parents described the information found online as distressing, especially if low life expectancy was stated (Boyse et al., 2014; DeLuca et al., 2011; Edwards et al., 2018; Jessup et al., 2016). In one article, a quarter of all participating parents were content with their searches (Edwards et al., 2018). Parents with higher levels of education were more likely to be satisfied (DeLuca et al., 2011; Edwards et al., 2018).

The main topics parents searched for were the condition itself, possible treatment options, expected quality of life, and life expectancy for the condition (Edwards et al., 2018). The sources regularly frequented were the respective condition associations

#### 3.4.2 Subcategory: focusing on actionable elements

Thirteen studies indicated that, over time, parents started to focus on modifiable areas that helped them to feel control and started to experience agency (Boyse et al., 2014; Carpenter et al., 2018; Chudleigh et al., 2016; DeLuca et al., 2011; Edwards et al., 2018; Howley et al., 2024; Jessup et al., 2016; Kutsa et al., 2022; Lebensburger et al., 2015; Priddis et al., 2010; Pruniski et al., 2018; Raspa et al., 2020, 2024).

One of the first active steps taken was parents’ management of their child’s condition at home (Howley et al., 2024). Parents first focused on the practical aspects of condition management, specifically symptom and treatment management at home (Boyse et al., 2014; Jessup et al., 2016; Lebensburger et al., 2015). Parents needed to learn new skills and gain condition-related knowledge to manage their child's condition at home, such as giving medicine, doing physiotherapy, adhering to a special diet, and taking more hygienic measures (Jessup et al., 2016; Pruniski et al., 2018).

Concurrently, parents took another active step by informing their family and friends about their child’s condition; while distressing for some, this step was helpful for others because it revealed aspects that were still unclear, and was a way of processing emotions (Chudleigh et al., 2016; Nielsen et al., 2022).

The new requirements of their child’s care, combined with no prior experience in condition management, were perceived as a stressor (Carpenter et al., 2018; DeLuca et al., 2011; Pruniski et al., 2018). To manage this, some parents accepted support from their family and friends, mainly for practicalities such as taking care of siblings and household duties (Nielsen et al., 2022). Parents with support from family and friends appeared to adapt better to the changes in their lives (Pruniski et al., 2018). However, this was only the case if parents felt that the people supporting them were capable; otherwise, it put more pressure on parents and caused some to worry about losing control (Carpenter et al., 2018).

Over time, condition management by parents progressed from learning to handle all tasks towards being able to gradually implementing treatment-related routines and adapting their daily family lives in a step-by-step manner (Kutsa et al., 2022). This seemed to be an ongoing process, because parents stated that they had to readjust their condition management practices and routines (Priddis et al., 2010), and went beyond the first 6 months post-diagnosis, as one parent explained in Pruniski et al. (2018):

*“I feel like that first year was probably the hardest, because it was the first time for everything. […] once we got through the first year […] it becomes a little easier […] So now our day to day is just a normal day to day.”*

Focusing on activities around condition management, their child’s treatment and well-being, and starting new routines in their daily life was a common way for parents to handle the situation and helped them manage uncertainties (Kutsa et al., 2022). Additionally, parents emphasised the importance of focusing on the moment (Priddis et al., 2010; Pruniski et al., 2018). Otherwise, they might lose their thoughts and emotions, as one mother explained in Pruniski et al. (2018).

*‘…You just kind of go down a spiral of what ifs and further and further until finally, you have to say 'yes, but right now she is ok'‘*

Another activity that helped parents to manage was reaching out to other parents of children with the same conditions. This led to reassurance for some parents (Boyse et al., 2014; Chudleigh et al., 2016; Edwards et al., 2018; Kutsa et al., 2022; Pruniski et al., 2018; Raspa et al., 2020).

#### 3.4.3 Subcategory: specialized HCP as point of contact and the preferred information source

Eleven of the included studies pointed out that when parents were suddenly confronted with the diagnosis of a chronic condition of their child, most of them had no experience in a healthcare setting. Parents felt that they were facing a new world (Chudleigh et al., 2016; DeLuca et al., 2011; Howley et al., 2024; Jessup et al., 2016; Lebensburger et al., 2015; Nielsen et al., 2022; Priddis et al., 2010; Pruniski et al., 2018; Raspa et al., 2020) and had to learn a new language (Boyse et al., 2014; Jessup et al., 2016). Most parents were highly motivated to learn more about their child’s condition; they felt empowered by the information given to them by HCPs and emphasised how ‘knowledge is power’ (Lebensburger et al., 2015; Nielsen et al., 2022; Pruniski et al., 2018). Regarding information materials, the main challenge was that each person has individual needs corresponding to the depth and amount of information provided (Edwards et al., 2018; Jessup et al., 2016).

The most valuable format to convey information was in a face-to-face setting, which allowed enough time to answer parents’ questions (Edwards et al., 2018; Jessup et al., 2016). Most parents wished for additional information (Edwards et al., 2018; Howley et al., 2024; Jessup et al., 2016; Nielsen et al., 2022), in print or available online (Edwards et al., 2018; Jessup et al., 2016). Among topics that were suggested for inclusion, the most important was information regarding the diagnosis, prognosis, condition itself, and genetics (Edwards et al., 2018). Others found that the information provided by patient organisations or associations was also helpful (Chudleigh et al., 2016; Edwards et al., 2018; Jessup et al., 2016).

Eight of the included studies indicated that first-contact with HCPs had an impact on the level of trust families built with HCPs later on (Chudleigh et al., 2016; DeLuca et al., 2011; Edwards et al., 2018; Howley et al., 2024; Jessup et al., 2016; Kutsa et al., 2022; Lebensburger et al., 2015; Nielsen et al., 2022). In addition, it has been shown to be crucial that the first notification and appointment at a clinic be with a specialist (Chudleigh et al., 2016; Howley et al., 2024; Lebensburger et al., 2015). Follow-up calls were equally important a few hours after the first notification and days after the first clinic visit (Nielsen et al., 2022). This led to stronger and more positive relationships between parents and HCPs (Nielsen et al., 2022). Parents felt less anxious and calmer after being given time to ask questions (DeLuca et al., 2011; Edwards et al., 2018; Howley et al., 2024; Kutsa et al., 2022; Nielsen et al., 2022). In general, parents felt some relief, especially if they perceived that the HCP had the situation under control (Nielsen et al., 2022).

#### 3.4.4 Sub subcategory – learning practical aspects of condition management at home

Practical information was reported to be useful, especially at the beginning (Edwards et al., 2018). One parent suggested handing out a ‘survival guide’ to parents after the diagnosis of their child (Boyse et al., 2014). This practical information should include content covering symptoms, complications, and instructions on condition management at home with specific statements on how to perform the necessary tasks (i.e.: giving medication) that they needed to start immediately (Boyse et al., 2014; Jessup et al., 2016; Lebensburger et al., 2015). Parents wished to receive information in print and in an online formats, ideally with illustrations (Boyse et al., 2014). Furthermore, parents wished to be informed on what they should expect in the near future and trustworthy sources for further information (Jessup et al., 2016). Also, focusing on tasks of condition management at home gave parents a sense of control (Jessup et al., 2016).

#### 3.4.5 Sub subcategory - more detailed information including aspects of family management

Parents preferred to obtain information over a longer time period (Jessup et al., 2016). In another study, parents’ need for accessible and understandable information was expressed throughout the course of the condition (Raspa et al., 2020). Parents’ information needs changed over the first weeks from performing the necessary aspects of condition management to wanting more detailed information on aspects concerning life outside the home (Jessup et al., 2016). Around 6 months post-diagnosis, parents appeared to start thinking about their social life again and wanted to be prepared for returning to a more normal daily life (Jessup et al., 2016). Additionally, parents expressed a need for information on factors they can actively influence as well as on financial aspects (Jessup et al., 2016).

## 4 Discussion

This IR highlights the experiences and information needs of parents during the unique trajectory of positive Newborn Screening results, which led to a diagnosis of a chronic condition in their neonate. The synthesised findings from 14 mostly qualitative studies of good to excellent quality highlighted the sudden onset of this trajectory. Parents were in shock and needed time to find an active approach to manage their child’s condition and their family life, for which they value access to trusted HCPs.

### 4.1 Experience

The synthesised articles suggested that initially, parents were in a state of shock and felt out of control. Our findings are in line with those of White et al. (2021), who stated that parents must first process the new situation. It is known that after the diagnosis of a chronic condition, parents are confronted with many distressing emotions like sorrow and grief for not having a ‘healthy’ child (Boardman and Clark, 2022; Pelentsov et al., 2015). In addition, parents must adjust to a new and different vision of their family's future (Boardman and Clark, 2022). An active stance towards living with the child’s condition is related to ‘feeling in control’ and reflects a problem-focused and proactive condition management strategy, as confirmed by prior research (Cousino and Hazen, 2013; Pelentsov et al., 2015; Piercy et al., 2019; Siddiq et al., 2016). Similarly, perceived competency in condition management and an understanding of the condition have been demonstrated to increase the health-related quality of life of parents of chronically ill children (Rautmann et al., 2023). Our finding of parental online information-seeking behaviour (OISB) seems to supplement information given by HCPs, which is seen as the preferred and most trusted source of information. Likewise, it fulfils an immediate and urgent information need during the waiting period before the first appointment at the hospital (Conway et al., 2022) and shortly after diagnosis (Gruebner et al., 2022). Seeking information is a way of regaining control in a situation with high uncertainty; it can help parents prepare for change and feel empowered (Dandy et al., 2024; Gruebner et al., 2022) and is motivated to understand and reassure (Powell et al., 2011). Moreover, searching for health information online has become a strategy because of the vast and diverse information that is available and accessible around the clock, which makes it convenient for patients - or in case of our review - parents to access (Gruebner et al., 2022; Jia et al., 2021; Powell et al., 2011).

Nonetheless, there is a discrepancy between HCP’s advice on not searching online and the behaviour that parents cannot stop themselves from enacting. To support parents better throughout the journey, it is important to understand online health information-seeking behaviours. This is in line with other authors emphasising that OISB is a reality, especially for younger patients and their families (Sørensen et al., 2012). HCPs are actively involved in providing advice and instructions (Jia et al., 2021).

### 4.2 Information needs

A well-covered topic in the synthesised articles is that HCPs are parents' preferred sources of information. This corresponds with previous studies showing that parents of children with chronic conditions, particularly rare conditions (Domaradzki and Walkowiak, 2024), often wish for more support and structured information from HCPs (Tluczek et al., 2022; White et al., 2021). Receiving structured information repeatedly from HCPs helped parents deal with their emotions and feel calmer (Dandy et al., 2024).

Additionally, parents seemed to appreciate informational material and educational programs, if available. Parents appeared to have a wide range of educational needs and preferences, aligning with prior findings (Nightingale et al., 2017; White et al., 2021). Parents felt that it was helpful to have the possibility of returning to written handouts (Ringnér et al., 2021) and receiving information in smaller portions over the course of time (White et al., 2021). In addition, parents valued receiving information from different health professionals, including practical information on how to manage their child’s condition (Conway et al., 2022); this is in line with the findings of this review. Rautmann et al. showed that well-informed parents rate their ability to manage their condition better and have better health-related quality of life (Rautmann et al., 2023).

The studies reveal a discrepancy between parents' need to understand the condition and their limited ability to absorb information due to emotional distress following a positive Newborn Screening result. This aligns with prior research showing parents attempt to position their child's condition on a 'severity spectrum' to understand its impact on family life, relationships, and goals.

(Piercy et al., 2019; Waxler et al., 2013; White et al., 2021; Wightman et al., 2019). Parents want to know the ‘worst case scenario’ of their child’s condition, to estimate the magnitude of the situation (Piercy et al., 2019), which is confirmed by the findings of this review. White et al. (2021) highlighted the importance of first assessing the parents’ capacity to understand the information given to them.

Additionally, early contact with specialists seems to have a positive effect on the relationships families form later with the HCP. If specialised HCPs were available, parents tended to trust their knowledge and judgment of the situation, which is in line with other studies (Dandy et al., 2024; Nygård and Clancy, 2018; Smith et al., 2015). Feeling heard by HCPs is a key factor (Baker and Claridge, 2022; Nygård and Clancy, 2018) and shows empathy, respect, and support for the interaction (Baker and Claridge, 2022; Dandy et al., 2024; Smith et al., 2015). And it promotes trust and therefore helps achieve a respectful relationship (Dandy et al., 2024; Nygård and Clancy, 2018; Smith et al., 2015), which was validated by this review. Negative experiences, such as unavailable specialists, permanently disrupted parent-HCP relationships (Conway et al., 2022). Research shows that delivering quality care from initial contact through diagnosis requires substantial HCP time (Conway et al., 2022), which is challenging given the global healthcare staff shortage (Michaeli et al., 2024). Additionally, geographic barriers make accessing specialized care difficult for some parents (Conway et al., 2022).

Although HCPs are parents' preferred information source, they often supplement this with online information. Technological advances present both challenges and opportunities: telemedicine can facilitate access to specialized care (Conway et al., 2022), and AI tools could free HCP time for patient interaction, which parents highly value (Haley et al., 2024). However, parents of hospitalized children emphasize the importance of human connection—only 36% were comfortable with computers answering most questions instead of people (Haley et al., 2024).

## 5 Strength and limitations

Our review included studies with different methodological approaches and combined them, which is a strength. However, it also had limitations. With the inductive approach chosen to analyse the extracted data, it was possible to develop a conceptual model. An IR methodology allows for conceptual and empirical work to be included, and therefore particularly suited to answer the research question even though no conceptual publication was identified (Toronto and Remington, 2020). A limitation is that one author conducted article selection and inclusion without screening pilot or inter-reviewer reliability assessment. We addressed this by discussing the selection process at multiple points with the last author. For quality appraisal, we used reporting guidelines to score not only item mention but also quality, which is not the gold standard but allowed us to apply consistent tools with one specific for mixed-methods studies. Of the 14 studies, 11 used a qualitative methodology, leading to an uneven distribution of methodological approaches. The qualitative approach of CCA allowed the results of the different designs to be combined equally despite the imbalance. Still, it must be assumed that the results have more qualitative focus; thus, for a quantitative interpretation of the findings, caution must be taken. We followed the methods and recommendations of Whittemore and Knafl (2005); for the synthesis, we used a CCA (Glaser, 1965), which is another strength. Although we applied the general principles of CCA, it cannot be ruled out that subjective views and experiences in this field may have influenced the synthesis. To minimize this bias, the analysis process was actively reflected on the different analysis steps were discussed with the last author. Most participants in the included studies were mothers. This leads to an overrepresentation of their views, whereas the fathers’ perspectives cannot be adequately or with certainty illustrated and concluded. Additionally, most geographically included studies were from English-speaking countries, and all were from developed countries. This is probably because Newborn Screening has mainly been established in developed countries; thus, this distribution of studies was to be expected.

Additionally, we only included articles in English, and there may have been articles on this topic that were overlooked. Although we followed a structured approach and searched five different search engines, there is still a possibility that suitable articles were excluded because of the differing wording of the leading search terms, which can vary in form. To minimize this bias, an information specialist was consulted on the search strategy.

## 6 Conclusions and implications for research and practice

To our knowledge, this is the first review synthesizing parent’s experiences and information needs following their newborn’s diagnosis after a positive Newborn Screening test across multiple conditions. Parents have a strong need for condition-specific information and support. Most included studies reported no standardized protocol for psycho-social support or information delivery, including counselling by specialized HCP from different professions. Additionally, adequate information materials - both online and in print - are necessary.

These findings relate to the United Nations’ Sustainable Development Goals (SDG) for 2030 (United Nations General Assembly, 2015). Improved psycho-educational support programs would help healthcare systems meet SDG 3 (“Good Health and Well-Being”), particularly point 3.8 on universal health coverage and access to quality essential health-care services. Inadequate support for families of affected neonates can lead to poor condition management at home and negatively impact both health outcomes and family well-being.

Our findings also connect to SDG 10 (“Reduce inequalities within and among countries”), as socioeconomic status and education level affect information preferences regarding amount, detail and format. Finally, SDG 4 (“Quality Education”) is relevant through the health literacy and educational support parents require to manage their child’s condition, as the post-diagnosis period involves acquiring illness knowledge and new skills. touched through health literacy and the educational/ informational support.

These findings help HCPs better understand parent’s information needs following an unexpected diagnosis after a positive Newborn Screening test. Because the diagnosis is sudden, parents need time to process it and their emotional responses. While parents have high information needs, HCPs must consider their limited capacity to absorb information due to shock. HCPs should provide structured information repeatedly, allow processing time and ample opportunity for questions. A follow-up call on the same day as notification of the positive Newborn Screening test is advisable. The HCP contacting parents should be a specialist or knowledgeable about the condition to answer all questions. Empathy and active listening build trusting, respectful relationships. Since parents commonly seek online health information, HCPs should provide specific recommendations and guidance.

Information materials should be provided at different timepoints in a structured, repeated manner including practical management instructions and illness knowledge. Followed by more detailed information supporting long-term family management.

## Declaration of generative AI and AI-assisted technologies in the manuscript preparation process

During the preparation of the revision of this work re-submitted in May 2026 the authors used DeepL write in order to proofread, spell-check and improve language and readability of the changed sections. After using this tool, the authors reviewed and edited the content as needed and take full responsibility for the content of the published article.

## Funding

This work was supported by the Nursing Science Foundation of Basel, Switzerland [grant numbers ID 3108-2022]. The sponsor had no role in the development of the study design; collection, analysis, or interpretation of the data; writing of the manuscript; or the decision to submit the manuscript for publication.

## CRediT authorship contribution statement

**Patricia Luck:** Writing – original draft, Visualization, Project administration, Methodology, Formal analysis, Conceptualization. **Jana Pachlopnik -Schmid:** Writing – review & editing, Supervision, Methodology, Conceptualization. **Rahel Naef:** Writing – review & editing, Supervision, Methodology, Conceptualization. **André Fringer:** Writing – review & editing, Supervision, Methodology, Conceptualization. **Susan Kirk:** Writing – review & editing, Supervision, Methodology, Conceptualization. **Anna-Barbara Schlüer:** Writing – review & editing, Supervision, Methodology, Formal analysis, Conceptualization.

## Declaration of competing interest

The authors declare the following financial interests/personal relationships which may be considered as potential competing interests:

Patricia Luck reports financial support was provided by Nursing Science Foundation Switzerland. If there are other authors, they declare that they have no known competing financial interests or personal relationships that could have appeared to influence the work reported in this paper.

## Figures and Tables

**Table:** 

**No**	**Search**	**No**	**Search**
1	parental [Title/ Abstract]	24	“Health Literacy” [Mesh]
2	maternal [Title/ Abstract]	25	“Information Sources” [Mesh]
3	paternal [Title/ Abstract]	26	“Information Seeking Behaviour” [Mesh]
4	families [Title/ Abstract]	27	“Education” [Mesh]
5	“parents” [Mesh	28	“education” [Subheading]
6	“Mothers” [Mesh]	29	“Coping Skills” [Mesh]
7	“fathers” [Mesh]	30	“Family Nursing” [Mesh]
8	“family” [Mesh]	31	impact [Title/ Abstract]
9	1 OR 2 OR 3 OR 4 OR 5 OR 6 OR 7 OR 8	32	burden [Title/ Abstract
10	Newborn screening [Title/ Abstract]	33	psychosocial factors [Title/ Abstract]
11	NBS [Title/ Abstract]	34	information [Title/ Abstract]
12	Neonatal screening [Mesh]	35	education [Title/ Abstract]
13	Neonatal screening [Mesh]	36	need* [Title/ Abstract]
14	diagnosis [Title/ Abstract]	37	coping [Title/ Abstract]
15	diagnosed [Title/ Abstract]	38	family management [Title/ Abstract]
16	diagnostic [Title/ Abstract]	39	family centred care [Title/ Abstract]
17	positive [Title/ Abstract]	40	care [Title/ Abstract]
18	diagnos* [Title/ Abstract]	41	support [Title/ Abstract]
19	14 OR 15 OR 16 OR 17 OR 18	42	experience [Title/ Abstract]
20	“Caregiver Burden” [Mesh]	43	experienced [Title/ Abstract]
21	“Psychosocial Intervention” [Mesh]	44	experiences [Title/ Abstract]
22	Psychosocial Functioning” [Mesh]	45	experience* [Title/ Abstract]
23	“Information Literacy” [Mesh]	46	uncertaint* [Title/ Abstract]
		47	20–26 combined with OR
		48	9 AND 19 AND 47

## Data Availability

The authors agree to share the data with the published article, they will be made available on Mendeley.

## Appendix

A: Search in Pubmed June 10th 2025

Search word list based on PEO framework:

Population:

(1) “parent*” or “mother*” or “father*”

Exposure:

(2) "Newborn screening" and “diagnos*”

Outcome:

(3) experience* or “impact” or “burden” or "psycho-social factors"

(4) “information needs” or “coping strategy” or "family management" or "family-centred care", “care” or “support”.

No filters or limits were added. Each database was searched individually.

## References

[bib0001] Baker K., Claridge A.M. (2023). I have a Ph.D. in my daughter”: mother and child experiences of living with childhood chronic illness. J. Child Fam. Stud..

[bib0002] Boardman F., Clark C. (2022). We’re kind of like genetic nomads’: parents’ experiences of biographical disruption and uncertainty following in/conclusive results from newborn cystic fibrosis screening. Soc. Sci. Med..

[bib0003] Boyse K.L., Gardner M., Marvicsin D.J., Sandberg D.E. (2014). It was an overwhelming thing»: parents’ needs after infant diagnosis with congenital adrenal hyperplasia. J. Pediatr. Nurs..

[bib0004] Brockow I., Nennstiel U. (2019). Parents’ experience with positive newborn screening results for cystic fibrosis. Eur. J. Pediatr..

[bib0005] Buchbinder M., Timmermans S. (2012). Newborn screening for metabolic disorders: parental perceptions of the initial communication of results. Clin. Pediatr. (Phila.).

[bib0006] Carpenter K., Wittkowski A., Hare D.J., Medford E., Rust S., Jones S.A., Smith D.M. (2018). Parenting a child with phenylketonuria (PKU): an interpretative phenomenological analysis (IPA) of the experience of parents. J. Genet. Couns..

[bib0007] Chudleigh J., Buckingham S., Dignan J., O’Driscoll S., Johnson K., Rees D., Wyatt H., Metcalfe A. (2016). Parents’ Experiences of receiving the initial positive newborn screening (NBS) result for cystic fibrosis and Sickle cell disease. J. Genet. Couns..

[bib0008] Chudleigh J., Chinnery H., Bonham J.R., Olander E., Moody L., Simpson A., Morris S., Ulph F., Bryon M., Southern K. (2020). Qualitative exploration of health professionals’ experiences of communicating positive newborn bloodspot screening results for nine conditions in England. BMJ Open.

[bib0009] Conway M., Vuong T.T., Hart K., Rohrwasser A., Eilbeck K. (2022). Pain points in parents’ interactions with newborn screening systems: a qualitative study. BMC Pediatr..

[bib0010] Cousino M.K., Hazen R.A. (2013). Parenting stress among caregivers of children with chronic illness: a systematic review. J. Pediatr. Psychol..

[bib0011] Currier R., Puck J.M. (2021). SCID newborn screening: what we’ve learned. J. Allergy Clin. Immunol..

[bib0012] Dandy S., Wittkowski A., Murray C.D. (2024). Parents’ experiences of receiving their child’s diagnosis of congenital heart disease: a systematic review and meta-synthesis of the qualitative literature. Br. J. Health Psychol..

[bib0013] DeLuca J.M., Kearney M.H., Norton S.A., Arnold G.L. (2011). Parents’ experiences of expanded newborn screening evaluations. Pediatrics.

[bib0014] Domaradzki J., Walkowiak D. (2024). Ultra-rare ultra-care: assessing the impact of caring for children with ultra rare diseases. Eur. J. Paediatr. Neurol..

[bib0015] Edwards D.J., Wicking K., Smyth W., Shields L., Douglas T. (2018). Information needs of parents of infants diagnosed with cystic fibrosis: results of a pilot study. J. Child Health Care : Prof. Work. Child. Hosp. Community.

[bib0016] El-Hattab A.W., Almannai M., Sutton V.R. (2018). Newborn Screening. Pediatr. Clin. North Am..

[bib0017] Fabie N.A.V., Pappas K.B., Feldman G.L. (2019). The current State of newborn screening in the United States. Pediatr. Clin. North Am..

[bib0018] Farrell M.H., La Pean Kirschner A., Tluczek A., Farrell P.M (2020). Experience with parent follow-up for communication outcomes after newborn screening identifies carrier status. J. Pediatr..

[bib0019] Fonseca A., Nazaré B., Canavarro M.C. (2014). Parenting an infant with a congenital anomaly: an exploratory study on patterns of adjustment from diagnosis to six months post birth. J. Child Health Care.

[bib0020] Glaser B.G. (1965). The constant Comparative method of qualitative analysis. Soc. Study Soc. Probl..

[bib0021] Gruebner O., Van Haasteren A., Hug A., Elayan S., Sykora M., Albanese E., Naslund J., Wolf M., Fadda M., Von Rhein M. (2022). Digital platform uses for help and support seeking of parents with children affected by disabilities: scoping review. J. Med. Internet Res..

[bib0022] Haley L.C., Boyd A.K., Hebballi N.B., Reynolds E.W., Smith K.G., Scully P.T., Nguyen T.L., Bernstam E.V., Li L.T. (2024). Attitudes on artificial intelligence use in pediatric care from parents of hospitalized children. J. Surg. Res..

[bib0023] Howley E., Soomann M., Kreins A.Y. (2024). Parental engagement in identifying information needs after newborn screening for families of infants with suspected athymia. J. Clin. Immunol..

[bib0024] Hummelinck A., Pollock K. (2006). Parents’ information needs about the treatment of their chronically ill child: a qualitative study. Patient. Educ. Couns..

[bib0025] Jessup M., Douglas T., Priddis L., Branch-Smith C., Shields L., AREST-CF (2016). Parental experience of information and education processes following diagnosis of their infant with cystic fibrosis via newborn screening. J. Pediatr. Nurs..

[bib0026] Jia X., Pang Y., Liu L.S. (2021). Online health information seeking behavior: a systematic review. Healthcare.

[bib0027] Knafl K.A., Deatrick J.A., Havill N.L. (2012). Continued development of the family management style framework. J. Fam. Nurs..

[bib0028] Kutsa O., Andrews S.M., Mallonee E., Gwaltney A., Creamer A., Han P.K.J., Raspa M., Biesecker B.B. (2022). Parental coping with uncertainties along the severe combined immunodeficiency journey. Orphanet. J. Rare Dis..

[bib0030] Lebensburger J.D., Grosse S.D., Altice J.L., Thierry J.M., Ivankova N.V. (2015). Understanding and improving health education among first-time parents of infants with sickle cell anemia in Alabama: a mixed methods approach. J. Pediatr. Hematol. Oncol..

[bib0031] Loeber J.G., Platis D., Zetterström R.H., Almashanu S., Boemer F., Bonham J.R., Borde P., Brincat I., Cheillan D., Dekkers E., Dimitrov D., Fingerhut R., Franzson L., Groselj U., Hougaard D., Knapkova M., Kocova M., Kotori V., Kozich V., Schielen P.C.J.I. (2021). Neonatal screening in Europe revisited: an ISNS perspective on the current State and developments since 2010. Int. J. Neonatal. Screen..

[bib0032] Loura D., Ferreira A.M., Romeiro J., Charepe Z. (2024). Health-illness transition processes in children with complex chronic conditions and their parents: a scoping review. BMC Pediatr..

[bib0033] Michaeli D.T., Michaeli J.C., Albers S., Michaeli T. (2024). The healthcare workforce shortage of nurses and physicians: practice, theory, evidence, and ways forward. Policy Polit. Nurs. Pract..

[bib0034] Moola S., Munn Z., Sears K., Sfetcu R., Currie M., Lisy K., Tufanaru C., Qureshi R., Mattis P., Mu P. (2015). Conducting systematic reviews of association (etiology): the Joanna Briggs Institute’s approach. Int. J. Evid. Based Heal..

[bib0035] Nielsen P.B., Olesen H.V., Jensen C.S. (2022). Being affiliated to a cystic fibrosis centre is important for parents’ everyday life. Acta Paediatr..

[bib0036] Nightingale R., Wirz L., Cook W., Swallow V. (2017). Collaborating with parents of children with chronic conditions and professionals to design, develop and pre-pilot PLAnT (the parent learning needs and preferences assessment tool). J. Pediatr. Nurs..

[bib0037] Nygård C., Clancy A. (2018). Unsung heroes, flying blind-A metasynthesis of parents’ experiences of caring for children with special health-care needs at home. J. Clin. Nurs..

[bib0038] Pelentsov L.J., Laws T.A., Esterman A.J. (2015). The supportive care needs of parents caring for a child with a rare disease: a scoping review. Disabil. Health Care.

[bib0040] Piercy H., Yeo M., Yap S., Hart A.R. (2019). What are the information needs of parents caring for a child with glutaric aciduria type 1?. BMC. Pediatr..

[bib0041] Powell J., Inglis N., Ronnie J., Large S. (2011). The characteristics and motivations of online health information seekers: cross-sectional survey and qualitative interview study. J. Med. Internet Res..

[bib0042] Priddis L., Dunwoodie J., Balding E., Barrett A., Douglas T. (2010). Paternal experiences of their children’s diagnosis of Cystic Fibrosis following newborn screening diagnosis. Neonatal Paediatr. Child Health Nurs..

[bib0043] Pruniski B., Lisi E., Ali N. (2018). Newborn screening for Pompe disease: impact on families. J. Inherit. Metab. Dis..

[bib0044] Raspa M., Kutsa O., Andrews S.M., Gwaltney A.Y., Mallonee E., Creamer A., Han P.K.J., Biesecker B.B. (2024). Uncertainties experienced by parents of children diagnosed with severe combined immunodeficiency through newborn screening. Eur. J. Hum. Genet..

[bib0045] Raspa M., Lynch M., Squiers L., Gwaltney A., Porter K., Peay H., Huston A., Fitzek B., Boyle J.G. (2020). Information and emotional support needs of families whose infant was diagnosed with SCID through newborn screening. Front. Immunol..

[bib0046] Rautmann L., Witt S., Theiding C., Odenwald B., Nennstiel-Ratzel U., Dörr H.-G., Quitmann J.H. (2023). Caring for a child with congenital adrenal hyperplasia diagnosed by newborn screening: parental health-related quality of life, coping patterns, and needs. Int. J. Env. Res. Public Health.

[bib0047] Ringnér A., Olsson C., Eriksson E., From I., Björk M. (2021). A moment just for me – parents’ experiences of an intervention for person-centred information in paediatric oncology. Eur. J. Oncol. Nurs..

[bib0048] Shoghi M., Shahbazi B., Seyedfatemi N. (2019). The effect of the Family-centered empowerment model (FCEM) on the care burden of the parents of children diagnosed with cancer. Asian Pac. J. Cancer Prev.: APJCP.

[bib0049] Siddiq S., Wilson B.J., Graham I.D., Lamoureux M., Khangura S.D., Tingley K., Tessier L., Chakraborty P., Coyle D., Dyack S., Gillis J., Greenberg C., Hayeems R.Z., Jain-Ghai S., Kronick J.B., Laberge A.-M., Little J., Mitchell J.J., Prasad C. (2016). Experiences of caregivers of children with inherited metabolic diseases: a qualitative study. Orphanet. J. Rare Dis..

[bib0050] Smith J., Cheater F., Bekker H. (2015). Parents’ experiences of living with a child with a long-term condition: a rapid structured review of the literature. Health Expect..

[bib62] Smith J.A., Osborn M. (2008). Qualitative psychology: A practical guide to research methods (S. 53–80).

[bib60] Sandelowski M. (2000). Whatever happened to qualitative description?. Res. Nurs. Health.

[bib61] Smith, J.A. (Hrsg.). (2001). Semi-structured Interviewing and Qualitative Analysis. In Qualitative psychology: A practical guide to research methods (1th edition). Sage.

[bib0051] Sørensen K., Van Den Broucke S., Fullam J., Doyle G., Pelikan J., Slonska Z., Brand H. (2012). Health literacy and public health: a systematic review and integration of definitions and models. BMC Public Health.

[bib0052] Thomas S., Ryan N.P., Byrne L.K., Hendrieckx C., White V. (2023). Unmet supportive care needs of families of children with chronic illness: a systematic review. J. Clin. Nurs..

[bib0053] Tluczek A., Ersig A.L., Lee S. (2022). Psychosocial issues related to newborn screening: a systematic review and synthesis. Int. J. Neonatal. Screen..

[bib0054] Toronto C.E., Remington R. (2020).

[bib0055] United Nations General Assembly (2015). https://sdgs.un.org/2030agenda.

[bib0056] Waxler J.L., Cherniske E.M., Dieter K., Herd P., Pober B.R. (2013). Hearing from parents: the impact of receiving the diagnosis of Williams syndrome in their child. Am. J. Med. Genet. A.

[bib0057] White A.L., Boardman F., McNiven A., Locock L., Hinton L. (2021). Absorbing it all: a meta-ethnography of parents’ unfolding experiences of newborn screening. Soc. Sci. Med..

[bib0058] Whittemore R., Knafl K. (2005). The integrative review: updated methodology. J. Adv. Nurs..

[bib0059] Wightman A., Zimmerman C.T., Neul S., Lepere K., Cedars K., Opel D. (2019). Caregiver experience in pediatric dialysis. Pediatrics.

